# Intraperitoneal malpositioning of an extracorporeal membrane oxygenation cannula: a case report

**DOI:** 10.1186/s13256-025-05663-8

**Published:** 2025-11-21

**Authors:** Alessandro De Cassai, Nicolò Sella, Francesco Zarantonello, Annalisa Boscolo, Giulia Aviani Fulvio, Paolo Navalesi

**Affiliations:** 1 Department of Medicine, University of Padua, Padua, Italy; 2 Anesthesia and Intensive Care Unit, University-Hospital of Padua, Padua, Italy; 3 Department of Cardiac, Thoracic, Vascular Sciences and Public Health, University of Padua, Padua, Italy

**Keywords:** Acute respiratory distress syndrome, Cannula malposition, Displacement, Extracorporeal membrane oxygenation, Pneumonia, ECMO complications, Ultrasound

## Abstract

**Background:**

Venovenous extracorporeal membrane oxygenation is an advanced life support modality used in cases of severe respiratory failure when conventional ventilation is no longer effective. While ultrasound and echocardiographic guidance have improved the safety of cannulation, malposition of extracorporeal membrane oxygenation cannulas remains a rare but potentially life-threatening complication. This case presents an unusual instance of intraperitoneal cannula misplacement during venovenous extracorporeal membrane oxygenation initiation, despite the use of standard imaging guidance techniques.

**Case presentation:**

A 39-year-old Italian woman with no significant prior medical history developed *Staphylococcus aureus* pneumonia that rapidly progressed to acute respiratory distress syndrome. She was intubated and managed with lung-protective mechanical ventilation and prone positioning. Despite these interventions, she developed refractory hypoxemia and hypercapnia, with a PaO₂/FiO₂ ratio dropping to 30 on 100% FiO₂. Venovenous extracorporeal membrane oxygenation support was initiated. Vascular access was obtained under surface ultrasound and transesophageal echocardiographic guidance. A 19-French return cannula was successfully inserted into the right internal jugular vein. A 25-French drainage cannula was introduced into the left femoral vein without resistance. However, instead of blood, a yellow translucent fluid returned from the femoral cannula, raising suspicion of malposition. The cannula was clamped and left in situ while an alternative cannula was inserted in the right femoral vein to commence extracorporeal membrane oxygenation support. Urgent contrast-enhanced computed tomography (CT) revealed that, although the guidewire had initially tracked correctly, a kink in its path led to the cannula entering the peritoneal cavity, forming a loop within the Douglas pouch. The misplaced cannula was safely removed without complications. The patient remained on extracorporeal membrane oxygenation for 11 days and required an additional 6 days of mechanical ventilation before successful extubation. She was eventually discharged without further issues.

**Conclusions:**

This case underscores that, even with appropriate imaging guidance, extracorporeal membrane oxygenation cannula malposition can still occur. A high index of suspicion, careful interpretation of procedural cues, and prompt diagnostic imaging are essential to prevent serious complications. Early recognition and intervention not only preserve the safety and efficacy of extracorporeal membrane oxygenation therapy but also can lead to favorable patient outcomes.

**Supplementary Information:**

The online version contains supplementary material available at 10.1186/s13256-025-05663-8.

## Background

Venovenous extracorporeal membrane oxygenation (VV-ECMO) is a lifesaving lung supportive therapy increasingly employed as a life-sustaining intervention for patients with severe respiratory failure that is unresponsive to conventional therapy. Its use has been notably supported by evidence from randomized controlled trials and observational studies, especially in the context of acute respiratory distress syndrome (ARDS) [1, 2], and although the indications for VV-ECMO are progressively expanding, its primary indications still include severe hypoxemia (PaO₂/FiO₂ ratio < 80 mmHg for > 6 h), uncompensated hypercapnia with acidemia (pH < 7.20), or excessive plateau pressures despite lung-protective ventilation [3].

ARDS is a common reason for initiating VV-ECMO, especially when the inflammatory response leads to refractory hypoxemia despite optimal ventilator settings and adjunctive measures such as prone positioning and neuromuscular blockade [4–6].

The procedure of ECMO cannulation—most commonly through femoral and internal jugular venous access—is a technically challenging procedure that must balance the urgency of support initiation with the need for procedural safety. The Seldinger technique, aided by ultrasound and transesophageal echocardiography, is now considered standard practice to reduce risks associated with blind cannulation, such as vascular trauma, incorrect placement, or extravascular migration [7, 8].

However, despite adherence to best practices, complications related to ECMO cannulation remain a significant concern. This report presents a rare and clinically instructive case of intraperitoneal misplacement of a femoral drainage cannula during VV-ECMO initiation in a patient with *Staphylococcus aureus*-induced ARDS. It highlights the importance of maintaining a high index of suspicion for cannulation-related complications—even in the presence of imaging guidance—and reinforces the value of early diagnostic imaging to prevent adverse outcomes.

## Case presentation

A 39-year-old Italian female patient developed *Staphylococcus aureus* pneumonia, which rapidly progressed to ARDS. She was intubated and received 3 days of lung-protective invasive mechanical ventilation in combination with prone positioning. Despite these measures, her respiratory function continued to deteriorate, resulting in severe hypercapnia and refractory hypoxemia, with a PaO₂/FiO₂ (P/F) ratio dropping to as low as 30 while on 100% FiO₂.

Given the severity of her condition and failure to respond to conventional therapy, VV-ECMO was initiated to provide advanced respiratory support.

During cannulation, vascular access was achieved using ultrasound guidance, while transesophageal echocardiography was employed to confirm proper placement of guidewires in both the right atrium (via the internal jugular vein) and the inferior vena cava (IVC) (via the femoral vein). Using the Seldinger technique, a 19-French return cannula was uneventfully inserted into the right internal jugular vein. A 25-French drainage cannula was then inserted via the left femoral vein without resistance.

While adequate blood return confirmed correct positioning of the jugular cannula, the left femoral cannula produced a translucent yellow fluid, raising immediate concern for malposition. The suspect cannula was promptly clamped and left in situ, and a second drainage cannula was successfully placed in the right femoral vein to allow ECMO support to begin without delay.

**Fig. 1:**
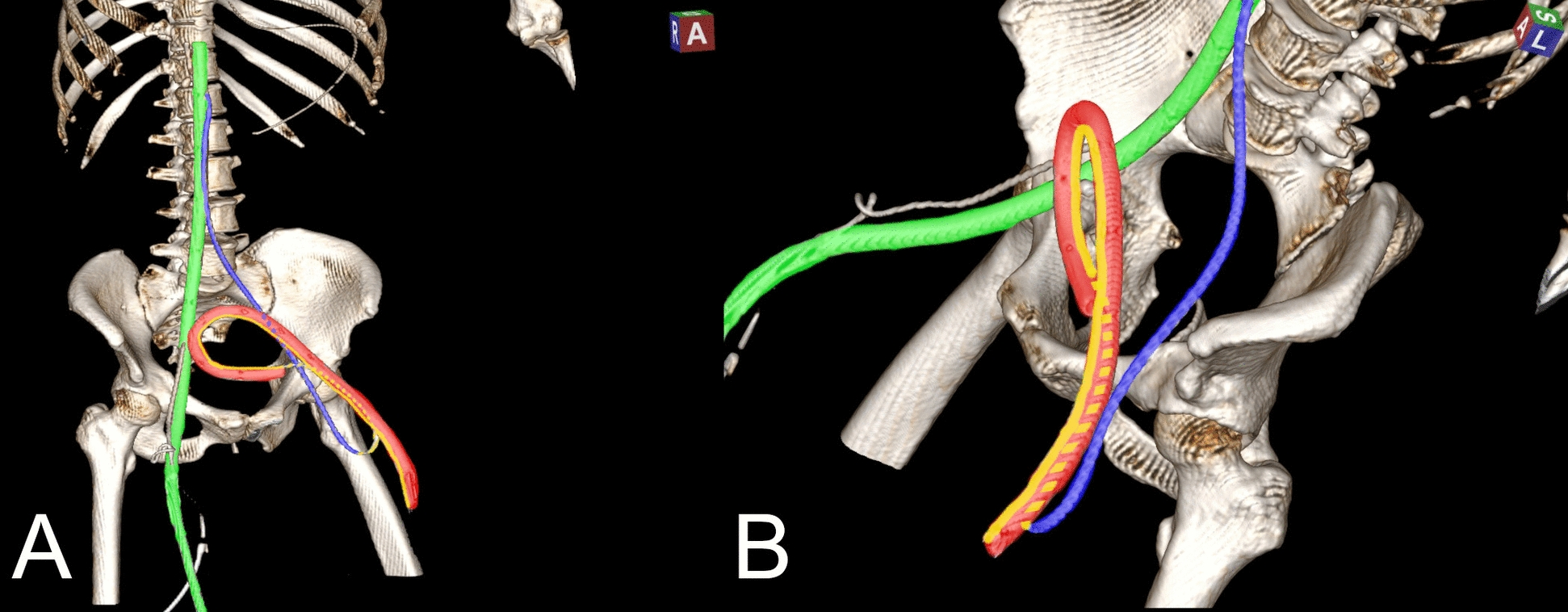
Three-dimensional reconstruction of the computed tomography scan. Panels **A** and **B** show two different view angles of the same picture. The computed tomography findings revealed that the right femoral cannula was properly placed intravascularly (green), while the left cannula was in the wrong location, intraperitoneally in the Douglas space (red). Interestingly, the guidewire of the misplaced cannula was in the correct intravascular position, and its trajectory can be traced from the femoral vein to the inferior vena cava (blue line). However, owing to wire kinking (as seen at the blue–yellow crossing point), an extravascular portion of the wire was also visible (yellow line, dashed in its path outside the cannula). Consequently, the cannula followed the wire’s extravascular path and entered the peritoneum

Prior to removing the left femoral cannula, an urgent contrast-enhanced computed tomography scan was performed to determine its exact location and path. The scan confirmed correct placement of both the femoral and jugular guidewires; however, the left femoral cannula was not within the femoral vein. Instead, it had deviated from the vessel, forming a loop and entering the peritoneal cavity, specifically within the Douglas pouch (rectouterine space). Imaging clearly demonstrated that, although the guidewire had initially followed the correct path from the femoral vein to the inferior vena cava, a kink in the wire had created an extravascular trajectory, probably owing to mechanical stress during the advancement of the ECMO cannula over the guidewire. As a result, the cannula followed this aberrant path into the peritoneum (Fig. 1, Supplementary Video 1).

The misplaced cannula was safely and uneventfully removed. The patient remained on VV-ECMO support for a total of 11 days, followed by an additional 6 days of mechanical ventilation. She was successfully extubated, subsequently transferred to the ward, and later discharged from the hospital.

Written informed consent was obtained from the patient for publication of this case report and any accompanying images.

## Discussion

V-V ECMO cannulation, while life-saving, carries a significant risk of mechanical complications, including vessel perforation, hematoma, hemorrhage, guidewire malposition, and—more rarely—extravascular cannula misplacement, which can result in severe outcomes if not promptly recognized [9]. Other complications include retroperitoneal bleeding, limb ischemia, or cannula migration due to patient movement or positioning [10].

Accurate placement of the guidewire is critical; a misdirected guidewire can lead the cannula into unintended locations, such as the right ventricle, resulting in life-threatening complications including cardiac perforation and tamponade [11]. Kinking of the guidewire or cannula is another well-documented issue, especially in pediatric and neonatal ECMO, often compromising drainage or circuit flow and necessitating urgent correction [12].

According to the Extracorporeal Life Support Organization (ELSO) guidelines for adult respiratory ECMO [3], optimal performance requires careful planning of cannulation, appropriate patient selection, and continuous monitoring of flow dynamics. A well-chosen cannula and guidewire combination—ensuring smooth transition between components and adequate stiffness—is essential to prevent kinking, malposition, or vessel trauma [3].

While imaging guidance is standard during cannulation, malposition may not always be immediately apparent, particularly with basic chest X-ray. Therefore, point-of-care ultrasound and contrast-enhanced CT, when suspicion arises, are invaluable tools for identifying misplacement early [13].

In the case presented, despite the use of both ultrasound and transesophageal echocardiography, we hypothesize that, during cannulation, despite no resistance being felt by the operator, the cannula caused the wire to kink before entering the vessel, and once kinked, the wire could no longer effectively guide the cannula into the femoral vein, ultimately leading the cannula into the peritoneal cavity owing to the mechanical stress during cannula advancement. This hypothesis is consistent with previous reports describing similar mechanisms leading to guidewire or cannula failure in the pediatric population [12, 14].

To our knowledge, this is the first reported case of intraperitoneal cannula misplacement during VV-ECMO in the context of ARDS; nonetheless, other ECMO cannula misplacements have been previously documented, as described in Table 1.

**Table 1:** Previously reported ECMO cannula malposition sites

Displacement site	Clinical presentation	Diagnosis and management
Patent foramen ovale [15]	Malposition of the veno-arterial ECMO drainage cannula in the patent foramen ovale of a patient with Eisenmenger’s syndrome. No clinical manifestations were reported	Through transesophageal echocardiography, the dislocation was promptly recognized and immediately corrected
Middle hepatic vein [9]	Several hours after extracorporeal support initiation, ECMO flow became unstable. Bedside radiography and ultrasound (US) revealed that the tip of the venous drainage cannula was dislocated in the middle hepatic vein from IVC	The cannula was repositioned at a depth of 36 cm, securing it within the IVC at nearly 2.7 cm form the right atrium
Right hepatic vein [16]	A CT scan performed at day 4 surprisingly showed that the venous cannula had entered the right hepatic vein without giving signs of liver injury or organ dysfunction	The cannula was removed only at day 9 after its incidental finding
Accessory hepatic vein [17]	8 days after ECMO support initiation, US examination showed that the venous cannula ended in the accessory hepatic vein. Despite such malposition, no disturbance in the ECMO venous return was observed	Given the hemorrhagic and infective risks, moving or replacing the cannula was considered a high-risk maneuver. Because of adequate venous drainage, the cannula repositioning was delayed until day 10

Risk factors associated with cannula malpositioning may be both patient-related and iatrogenic. Patient anatomical variations, for instance, can significantly increase the risk of cannula dislocation, as outlined by Li *et al.* in a case report describing the dislocation of an ECMO cannula in the middle hepatic vein [9]. Here, the authors claimed that the complication was likely determined by an enlarged hepatic vein of the patient and by the acute angle (77.78°) formed by the middle hepatic vein and the IVC. Under typical anatomical conditions, a catheter misplacement would have been less likely because of the generally obtuse angle between these two veins.

Operator-dependent factors may also predispose to cannula misplacement. In a second case report [17] describing a misplacement of the drainage ECMO cannula from the IVC to the accessory hepatic vein, the authors in fact suggested that the cannula might have dislocated during patient transition between beds.

However, as far as our case is concerned, none of the above-mentioned risk factors was identified.

## Conclusion

This case highlights that, even with the use of gold-standard imaging modalities such as ultrasound and transesophageal echocardiography, ECMO cannulation can still be associated with rare but potentially serious complications. Accurate guidewire placement does not guarantee correct cannula positioning, especially in the presence of anatomical variations or procedural factors such as wire kinking.

Early recognition of atypical findings—such as unexpected fluid return—and continue monitoring of the patient before, during, and after the procedure can prevent harm and facilitate timely correction. Ultimately, this case underscores the critical importance of vigilant procedural monitoring, rapid diagnostic assessment, and a high index of suspicion for malposition, all of which are essential to ensuring the safety and success of ECMO therapy.

## Supplementary Information

Supplementary Material 1.Three-dimensional reconstruction of the computed tomography scans.

## Data Availability

Not applicable.

## Abbreviations

ARDS: Acute respiratory distress syndrome
ECMO: Extracorporeal membrane oxygenation
ELSO: Extracorporeal Life Support Organization
CT: Computed tomography
US: Ultrasound
VV: Venovenous
IVC: Inferior vena cava
